# Effects of train speed and passenger capacity on ground vibration of underground suburban railways

**DOI:** 10.1038/s41598-024-60782-4

**Published:** 2024-05-08

**Authors:** Shusen Cao, Dong Li, Zhimin Li, Hongkai Wang, Jili Yin, Chao Chen, Futong Wang

**Affiliations:** 1 Taizhou Taizhong Rail Transit Co., Ltd., Taizhou, 318014 Zhejiang China; 2 School of Civil Engineering and Architecture, Heilongjiang University, Harbin, 150080 China

**Keywords:** Power spectrum, Ground vibrations, Peak acceleration, Suburban railway systems, Train velocity, Civil engineering, Environmental sciences

## Abstract

This study aims to explore the optimal driving speed for ground vibration in suburban railway underground sections. We focused on the ground surface of suburban railway underground sections and developed a 3D finite element dynamic coupling model for the tunnel–soil system. Subsequently, considering factors such as train speed and passenger load, we analyzed the propagation characteristics of ground vibration responses in urban railway underground sections. The research results indicate a significant amplification phenomenon in the peak power spectrum of measurement points near the tunnels in underground sections. The high-frequency components of the power spectrum between measurement points are noticeably higher between the two tunnels. Furthermore, as the train speed increases, this amplification phenomenon becomes more pronounced, and the power spectrum of each measurement point mainly concentrates on several frequency bands, with the amplitude of the power spectrum near the prominent frequencies also increasing. However, when the train speed is between 100 and 120 km/h, the impact on the amplitude of the power spectrum at measurement points above the running tunnel is minimal. Additionally, the amplitude of the middle-to-high frequency components in the power spectrum increases with the increase in passenger numbers. The impact on the peak acceleration amplitude at each measurement point is minimal when the train speed is 80 km/h or below. However, once the train speed exceeds 80 km/h, the peak acceleration amplitude above the running tunnel rapidly increases, reaching its maximum value at 113 km/h, and then gradually decreasing.

## Introduction

The metro, a popular mode of underground transportation, is extensively utilized in the modernization of large cities due to its convenience, safety, and environmental benefits^1,2^. As of December 31, 2022, mainland China had commissioned urban rail transit lines totaling 10,291.95 km in length, with subways comprising 8012.85 km of this network, equating to 77.86%^3^. As the rail network continues to expand and train speeds increase, the associated environmental vibration issues become increasingly severe^4^. Vibrations resulting from the prolonged operation of subways have a detrimental impact on the durability of nearby buildings and directly affect the daily lives of residents in these buildings^5–7^. At present, the prevalent methodologies for investigating subway vibrations comprise on-site measurements^8–14^, and model testing^15^. Theoretical analyses^16,17^, and numerical simulations^18–24^. The efficacy of on-site measurements in studying subway vibrations is constrained by the limitations of surrounding sites and instrumentation, leading to challenges in addressing certain issues and in achieving reproducibility. Model testing, while comprehensive, incurs higher costs and similarly struggles with reproducibility. Currently, numerical simulation methods have gained widespread application due to their versatility. Standard approaches for simulating subway vibrations encompass empirical, analytical, and numerical methods^25^. Within the realm of numerical methods, the finite element method is distinguished by its high simulation accuracy. It is frequently employed to forecast the vibration response in more intricate systems and real-world projects^26–29^. Ding et al.^30^ employed a finite element model to analyze the dynamic response of the area surrounding the subway. However, their study was limited to speeds below 80 km/h, and they only conducted a simple analysis of the Vertical ground speed decay for different vehicle speeds. Xiong et al.^31^ developed a finite element model of tunnel–soil–building to analyze the impact of two different vibration sources on human comfort. However, it is noteworthy that their study did not consider the effects of train speed and train load on ground vibration. Zhou et al.^32^ investigated the dynamic response of structures near subway operations using a three-dimensional finite element coupled model, which included the train-railway-building-foundation. However, it is worth noting that their study only considered the effects of train operation at speeds below 80 km/h. Ma et al.^33^ constructed a three-dimensional finite element model of the track–tunnel–foundation–building system using the finite element software Abaqus. However, it is noteworthy that their study only explored the vibration characteristics of buildings along the high-speed train lines in soft soil areas, without considering the impact on ground vibration in the surrounding environment. Tian et al.^34^ established a two-dimensional plane model using the finite element software Abaqus to primarily study the vibration response of the underframe structure of trains at different speeds. However, it is worth noting that their study did not consider the propagation characteristics of vibrations on the ground surface, and only analyzed the vibration response of peak acceleration, lacking in-depth research on the propagation of vibrations on the ground surface and other frequency domain characteristics. Liu et al.^35^ and Zhang et al.^36^ established three-dimensional finite element dynamic models, focusing on analyzing the influence of the elastic modulus of the tunnel foundation and the depth of the tunnel on ground vibration. However, it is worth noting that their studies only analyzed the effects of different factors on the Z vibration level, without considering the impact on frequency domain characteristics. Xu^37^ established a dynamic finite element model of the “subway–tunnel–soil” system in his study, aiming to investigate the effects of various factors on ground vibration induced by the subway. However, it is worth noting that in their research, the values of train speed parameters were all below 80 km/h, and only the effects of various factors on the acceleration time history were considered, without involving an analysis of frequency domain characteristics. In their discussion, Verhas^38^ and Kurzweil et al.^39^ address the understanding of vibration propagation rules induced by trains or subways operating on ground level. Wang et al.^40^ developed an explicit time-domain three-dimensional (3D) finite element (FE) model to study ground vibration caused by trains. They compared soil responses induced by different numbers of vehicles, but it is worth noting that they did not investigate the differences in ground vibration under different train speeds. Gao et al.^41^ used a 2.5D finite element method (FEM) to study the dynamic response of unsaturated ground under the moving load of high-speed trains. Their research findings indicate that for both high-speed and low-speed trains near the center of the track, ground displacement rapidly attenuates at nearly the same rate.

While there has been extensive research on environmental vibrations around major transportation arteries and subway systems, studies focusing on the propagation characteristics of ground-level environmental vibrations in underground sections of suburban railways are relatively scarce. Specifically, research on the propagation characteristics of ground-level environmental vibrations caused by adjacent tunnels in underground sections of suburban railways is particularly lacking. Unlike typical urban rail transit systems, urban rail transit trains operate at higher speeds and have stricter construction requirements. Therefore, studying the ground-level environmental vibrations caused by suburban railways presents certain challenges. In this study, a three-dimensional finite element dynamic coupling model of the underground section of suburban railways and the ground environment was established using the finite element method to investigate the ground-level environmental vibrations caused by suburban railways. In the study of environmental vibrations caused by rail transit, determining the load forces generated during train operation has always been a challenge for many researchers. In traditional empirical methods, only three rail vibration peaks are considered. We expanded the rail vibration peaks to "i" peaks, which is more in line with actual conditions. The interaction forces between the wheel and rail were determined using optimization theory methods, and a three-dimensional finite element dynamic coupling model of the tunnel–soil structure was established to simulate and analyze the ground vibration response in the underground section of Taizhou suburban area. Additionally, this study also explores the propagation response of ground vibrations under different passenger loads and train speeds.

### Numerical modeling

#### Train load simulation

Train loading presents a significant challenge for researchers in geotechnical earthquake engineering. Li et al.^42^ employed the superposition of fluctuations to represent the load of multiple wheelsets using the Fourier series form. Nevertheless, since train loads are transmitted through rails and sleepers, which exhibit periodicity, they can be characterized by forces that encapsulate their periodic nature^43^.

Extensive theoretical research and experimental studies conducted at the British Railway Technology Center have demonstrated^44^ that the primary factors contributing to vertical wheel-rail forces include various forms of unevenness and localized wear on the wheel rim, among others. Furthermore, the vertical wheel-rail force predominantly manifests within three frequency ranges: (1) from 0.5 to 10 Hz, primarily resulting from the relative motion between the train body and the suspension system; (2) between 30 and 60 Hz, attributed to the rebound effect of the unsprung wheel mass on the rails; and (3) from 100 to 400 Hz, owing to the movement of the rails being impeded by the contact ground surface of the wheels and rails. The obtained measurements indicate that the wheel-rail forces are more pronounced in the 0–60 Hz range, whereas the higher frequency range predominantly influences the dynamic response of the car body.

Drawing upon existing research and data, an excitation force function can be utilized to model the dynamic loading of a train. This model encompasses static loads and a series of sinusoidal function iterations^45^.

1 $$ F\left( t \right) = P_{0} + P_{1} \sin \left( {\omega_{1} t} \right) + P_{2} \sin \left( {\omega_{2} t} \right) + P_{3} \sin \left( {\omega_{3} t} \right) $$

The formula, *P*_0_ represents the static load of the wheel; *P*_1_, *P*_2_, corresponds to the peak values of the vibrational loads associated with the circular frequency of rail vibrations; and *t* denotes time.

However, given that merely three vibrational loads are inadequate for a comprehensive simulation of the actual subway vibration scenario, Eq. (1) has been optimized. The enhanced dynamic loads are expressed as follows

2 $$ F\left( t \right) = P_{0} + \mathop \sum \limits_{i = 1}^{n} P_{i} \sin \left( {\omega_{i} t} \right) $$

here *n* represents the number of distinct rail vibration circular frequencies selected, and *P*_*i*_ signifies the peak value of the vibration corresponding to the *i*th rail vibration circular frequency.

Assuming the unsprung mass of the train is *M*_*0*_, the corresponding amplitude of the vibration load can be expressed as follows.

3 $$ P_{i} = M_{0} a_{i} \omega_{i}^{2} $$

The circular frequency is determined using the equation* M*_0_ = 750 kg, where *a*_*i*_ represents one of the typical vector heights associated with the respective wavelength in Table 1^46^.

**Table 1:** UK track geometry disturbance management values.

Control condition	Wavelength (m)	Sagittarius (mm)
Based on ride smoothness (I)	50	16
	20	9
	10	5
Dynamic surcharge loads affecting the routes (II)	5	2.5
	2	0.6
	1	0.3
Waveform abrasion (III)	0.5	0.1
	0.05	0.005

The circular frequency is calculated as follows:

4 $$ P_{i} = M_{0} a_{i} \omega_{i}^{2} $$

here *v* denotes the train’s running speed, and *L*_*i*_ represents the typical wavelength in the three scenarios (I, II, and III) as outlined in Table 1.

Following the field investigation, it was determined that the average speed of the underground section of the Taizhou City Regional Railway is 60 km/h. The train’s total length is approximately 92 m, with an axle weight of 16.5 t, comprising four cars. The tunnel depth is measured at 14.7 m, with an outer diameter of 8500 mm and an inner diameter of 7700 mm.

#### Determination of damping

The overall model’s power balance equation is presented as follows^47,48^:

5 $$ \left[ M \right]\left\{ {\ddot{u}} \right\} + \left[ C \right]\left\{ {\dot{u}} \right\} + \left[ K \right]\left\{ u \right\} = \left\{ {F\left( t \right)} \right\} $$

here $\left[ M \right]$ represents the mass matrix; $\left[ C \right]$ denotes the damping matrix; $\left[ K \right]$ signifies the stiffness matrix; $\left\{ {F\left( t \right)} \right\}$ corresponds to the time-dependent load; $\left\{ {\ddot{u}} \right\}$ is the acceleration vector, $\left\{ {\dot{u}} \right\}$ the velocity vector, and $\left\{ u \right\}$ the displacement vector. The accuracy of the damping matrix is crucial as it directly influences the vibration response of the model. In the transient analysis of underground geotechnical media structures, damping primarily manifests through the material's internal friction and dissipation. Consequently, Rayleigh damping is commonly employed for calculating the damping matrix. Rayleigh damping theory simplifies the damping matrix into a linear combination of the mass matrix $\left[ M \right]$ and the stiffness matrix $\left[ K \right]$ ^49^.

6 $$ \left[ C \right] = \alpha \left[ M \right] + \beta \left[ K \right] $$

where $\alpha$ is the viscous damping component and $\beta$ is the hysteresis or solid or stiffness damping component.

By the orthogonal condition of the vibration mode, the relationship between the coefficients $\alpha$ and $\beta$ to be determined and the modal damping ratio must fulfill the following equation:

7 $$ \zeta = \frac{\alpha }{2\omega } + \frac{\beta \omega }{2},\quad 0 \le \omega \le \omega_{max} $$

The Rayleigh damping coefficients $\alpha$ and $\beta$ in Eq. (6) can be determined by considering the intrinsic frequencies of two vibration modes, $\omega_{i}$ and $\omega_{j}$, along with their corresponding damping ratios $\zeta_{i}$ and $\zeta_{j}$. These values are then substituted into Eqs. (8) and (9), respectively, followed by solving the resultant system of equations.

8 $$ \alpha = \frac{{2\left( {\zeta_{j} \omega_{i} - \zeta_{i} \omega_{j} } \right)}}{{\omega_{i}^{2} - \omega_{j}^{2} }}\omega_{i} \omega_{j} $$

9 $$ \beta = \frac{{2\left( {\zeta_{i} \omega_{i} - \zeta_{j} \omega_{j} } \right)}}{{\omega_{i}^{2} - \omega_{j}^{2} }} $$

In this model, the values are set as follows: $\omega_{i} = {3}{\text{.142}}$, $\omega_{i} = {3}{\text{.142}}$, $\zeta_{i} = {48}{\text{.16\% }}$, and $\zeta_{i} = {24}{\text{.08\% }}$.

In real-world environments, machine vibrations and train-induced disturbances are unavoidable, and the complexity of the soil adds to the modeling challenges. Given these factors, we made several assumptions when establishing the model. We assumed that there were no other sources of disturbance during train operation, and we simplified the soil to a homogeneous condition. While these assumptions simplify the model, they help reduce the complexity of data collection and the large number of finite elements required.

The computational domain of the finite element model is 200 m wide, 100 m long, and 30 m deep. Ten-node elements are used to simulate the soil. Viscoelastic boundary conditions are applied to the model boundaries, and a total of 122,206 soil elements are simulated. During this process, we considered a fixed wheelbase of 2 m and a car length of 13 m. The time interval for dynamic simulation is set to 0.005 s, with a simulation duration of 15 s.

#### Soil parameters of the model

**Figure 1:**
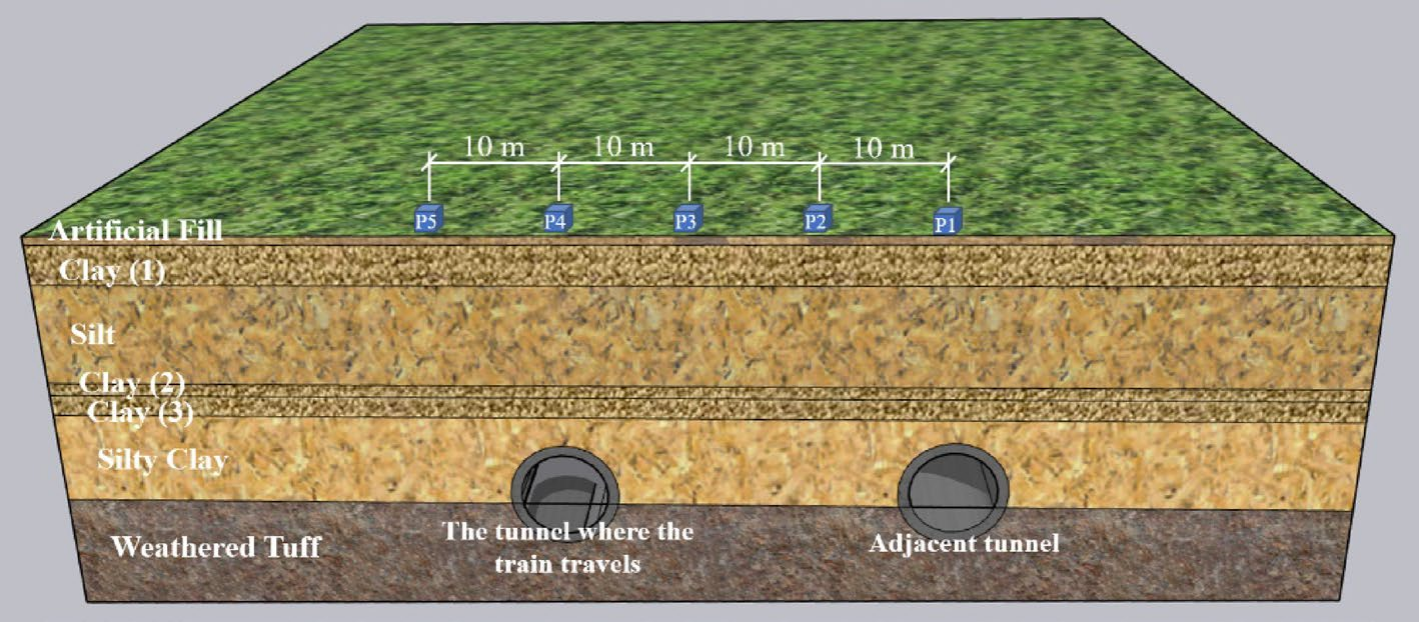
Schematic diagram of measuring point layout.

The research team conducted field observations of ground surface vibrations in the underground section of the Taizhou Suburban Railway, particularly in the vicinity of neighboring tunnels. The layout of the measurement points is depicted in Fig. 1. The soil parameters for the model, as presented in Table 2, were derived from geological exploration data.

**Table 2:** Calculation parameters of the soil layer in the underground section of the Taizhou suburban railway.

Soil layer	Soil thickness (m)	Shear wave (m s^−1^)	Densities (kN m^−3^)	Poisson’s ratio	Porosity ratio
Artificial fill	0.7	13.21	19	0.49	0.5
Clay(1)	3.2	30.56	18.7	0.49	0.5
Silt	8.1	24.66	17.1	0.49	0.5
Clay(2)	1	36.78	19.6	0.49	0.5
Clay(3)	1.7	51.74	18.7	0.49	0.5
Silty clay	7.4	62.89	19.6	0.49	0.5
Weathered tuff	7.9	97.99	24	0.49	0.5

#### Soil parameters of the model

To study the propagation characteristics and attenuation properties of ground vibration in the underground sections of suburban railways, we installed five measurement points above the tunnels, as shown in Fig. 1, depicting their relative positions to the tunnels. Despite the tunnels being adjacent bidirectional tunnels, there is still a distance of nearly 30 m between the centerlines of the two tunnels.

**Figure 2:**
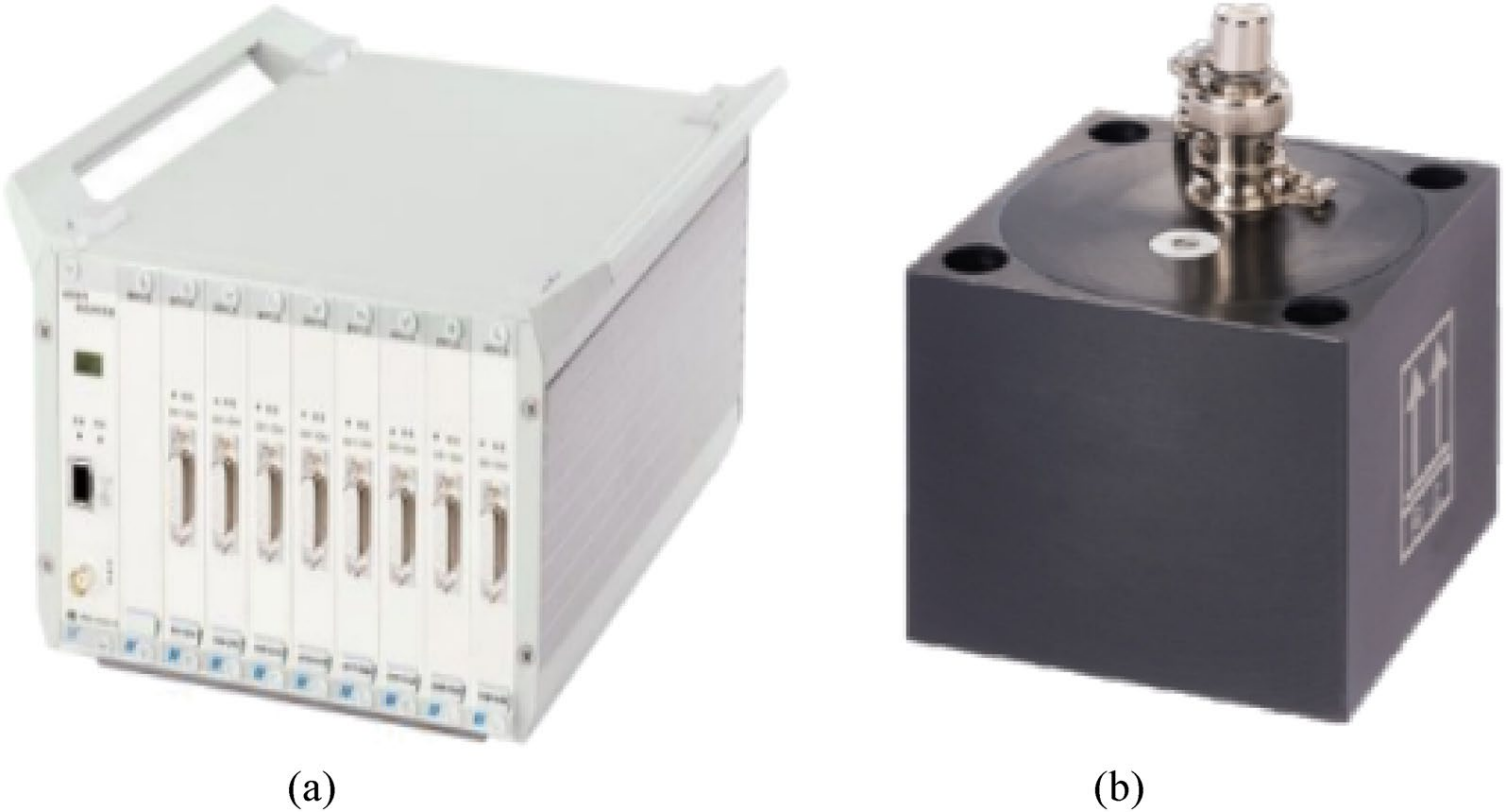
Data acquisition instrument. (**a**) DH5922D dynamic signal test and analysis system. (**b**)2D001 acceleration sensor.

We employed the Donghua 2D001 (Fig. 2a) accelerometer for data collection. The sensitivity of this sensor is 0.3 V s^2^/m, with a maximum range of 20 m/s^2^, and a frequency response range from 0.25 to 100 Hz. The data acquisition equipment used is the Donghua DH5922D (Fig. 2b) dynamic signal testing and analysis system.

**Figure 3:**
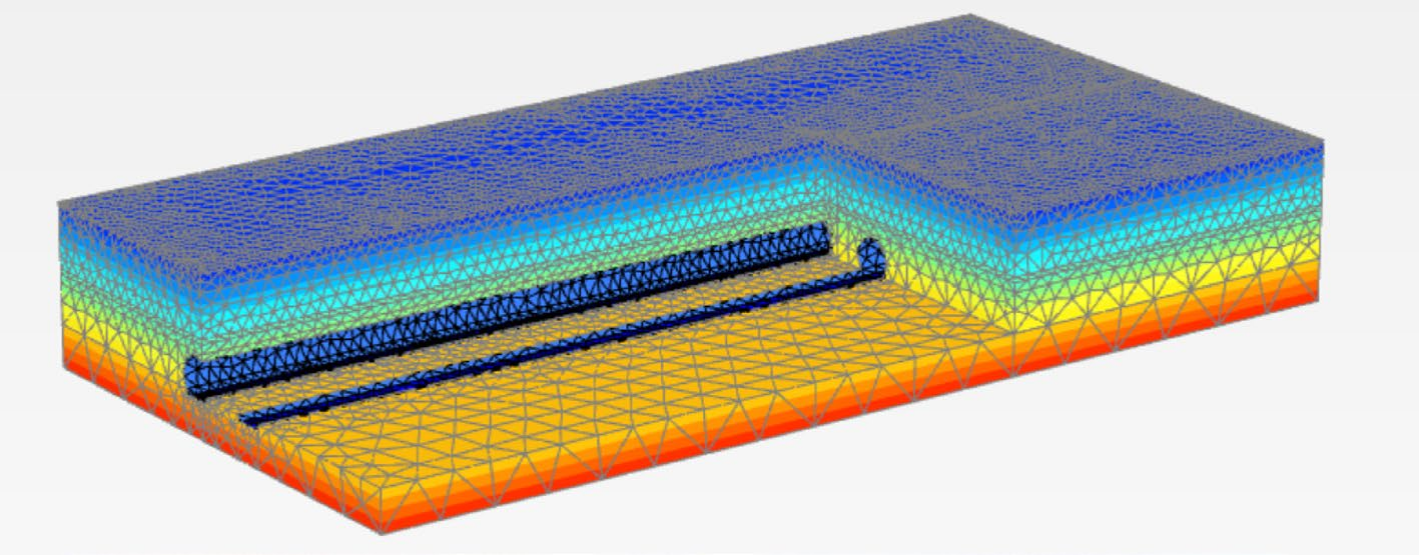
Schematic diagram of the finite element model.

**Figure 4:**
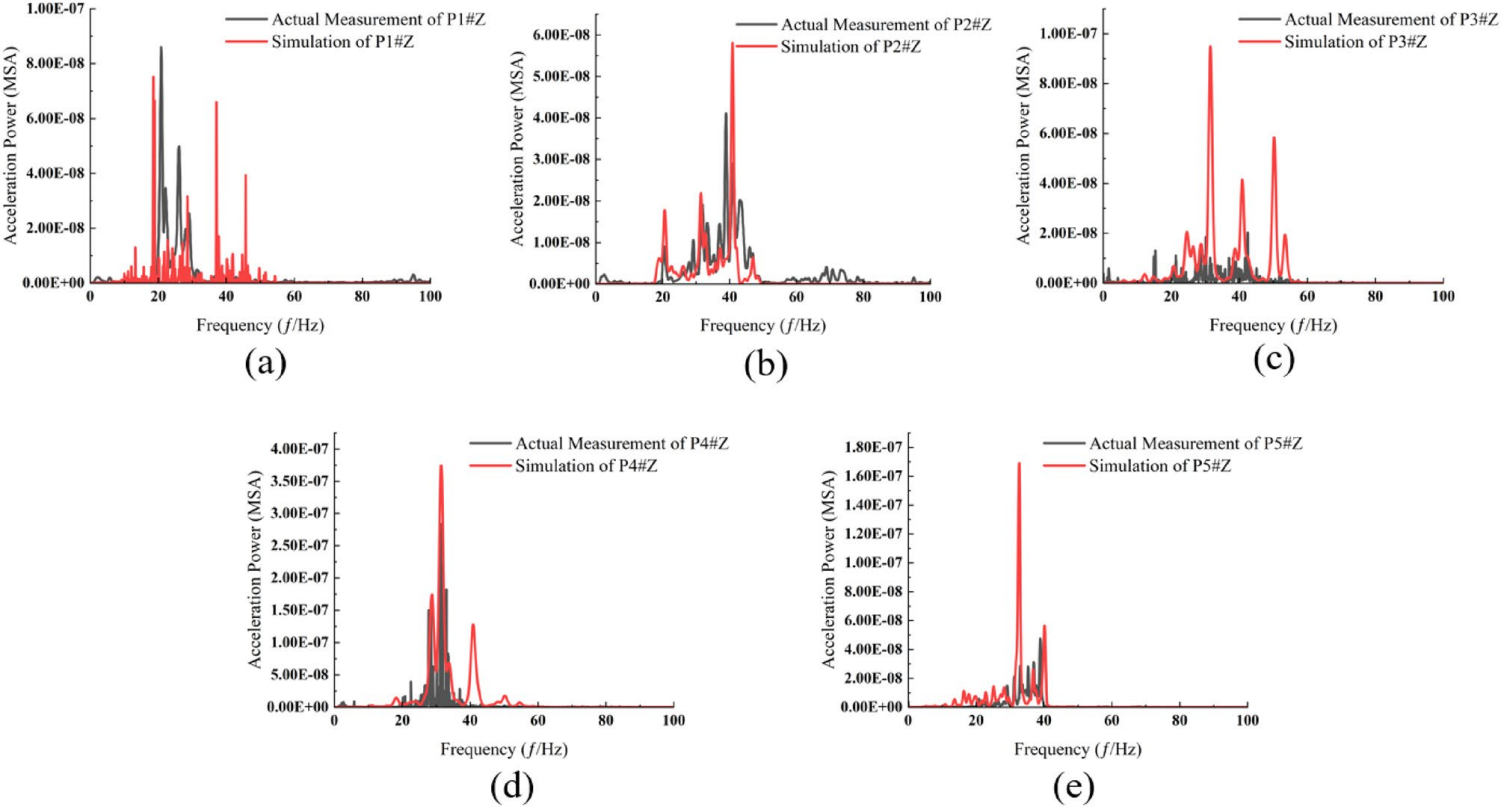
Comparison of calculated and measured spectrum values. (**a**) Measurement point P1. (**b**) Measurement point P2. (**c**) Measurement point P3. (**d**) Measurement point P4. (**e**) Measurement point P5.

**Figure 5:**
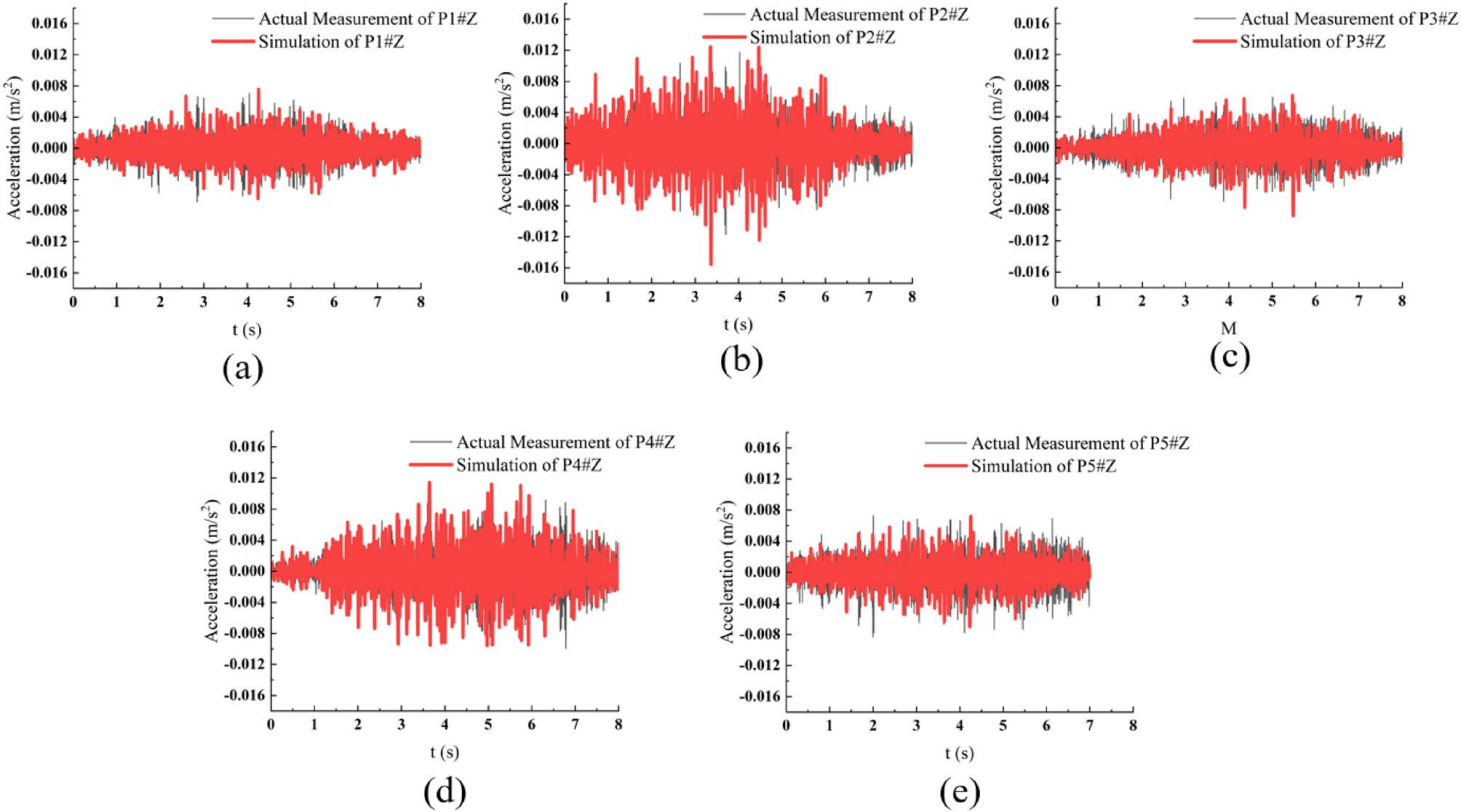
Comparison of calculated and measured spectrum values. (**a**) Measurement point P1. (**b**) Measurement point P2. (**c**) Measurement point P3. (**d**) Measurement point P4. (**e**) Measurement point P5.

To validate the reliability of the model, we set the train speed to 60 km/h. The load of the train was determined using an optimized empirical method and applied to the train track (Fig. 3). Subsequently, we extracted data from the acceleration time history obtained from the finite element model and performed a fast Fourier transform to obtain the acceleration power spectrum. Finally, we compared the simulated acceleration and acceleration power spectra obtained at each measurement point with the actual measured results. The comparison results, as shown in Figs. 4 and 5, exhibit good performance.

### Numerical results

#### Power spectrum analysis of ground vibration response to varying speed conditions

According to the “Technical Specification for Urban Rail Transit” (GB 50490-2009)^50^, this study categorizes train loads into four conditions: empty load, seated load, fixed load, and overloaded load, as explained below:

1. Empty load: Indicates that the train is without passengers and only carries its weight.
2. Seated load: Indicates that all seats on the train are occupied by passengers.
3. Fixed load: Refers to the calculation based on 6 persons/m^2^ after deducting the seating area per unit area inside the train.
4. Overloaded load: Refers to the calculation based on 9 persons/m^2^ after de-ducting the seating area per unit area inside the train, with each person weighing 60 kg and each car accommodating 60 passengers in the seating area.

In this paper, the optimal driving speed refers to the speed at which the changes in the power spectrum, Vibration acceleration levels, and maximum acceleration are relatively small as the train speed increases. In other words, when the train speed gradually increases to the optimal driving speed, the impact generated is less compared to when the speed is 60 km/h. Additionally, if the train needs to accelerate, the impact generated at the optimal driving speed is also smaller compared to the impact at other driving speeds.

This research examines the impact of trains operating at speeds from 60 to 140 km/h on ground vibrations within the underground area of the Suburban Railway, particularly under no-load conditions (Fig. 3).

The focus of this investigation is to assess the impact of these loading conditions on ground vibration in the underground sections of suburban railways, particularly in the speed range of 60–140 km/h. For ease of subsequent analysis, we categorize the frequency bands as follows: 0–20 Hz as low frequency, 20–40 Hz as mid-frequency, and above 40 Hz as high frequency. In-ground vibration research, low-frequency signals may arise from factors such as train operation, mechanical vibrations, or other low-frequency sources. Mid-frequency signals, in certain cases, may be caused by factors like train tracks, structural vibrations, or other mid-frequency sources. High-frequency signals may stem from sources such as machinery, high-speed motion, or other high-frequency sources.

**Figure 6:**
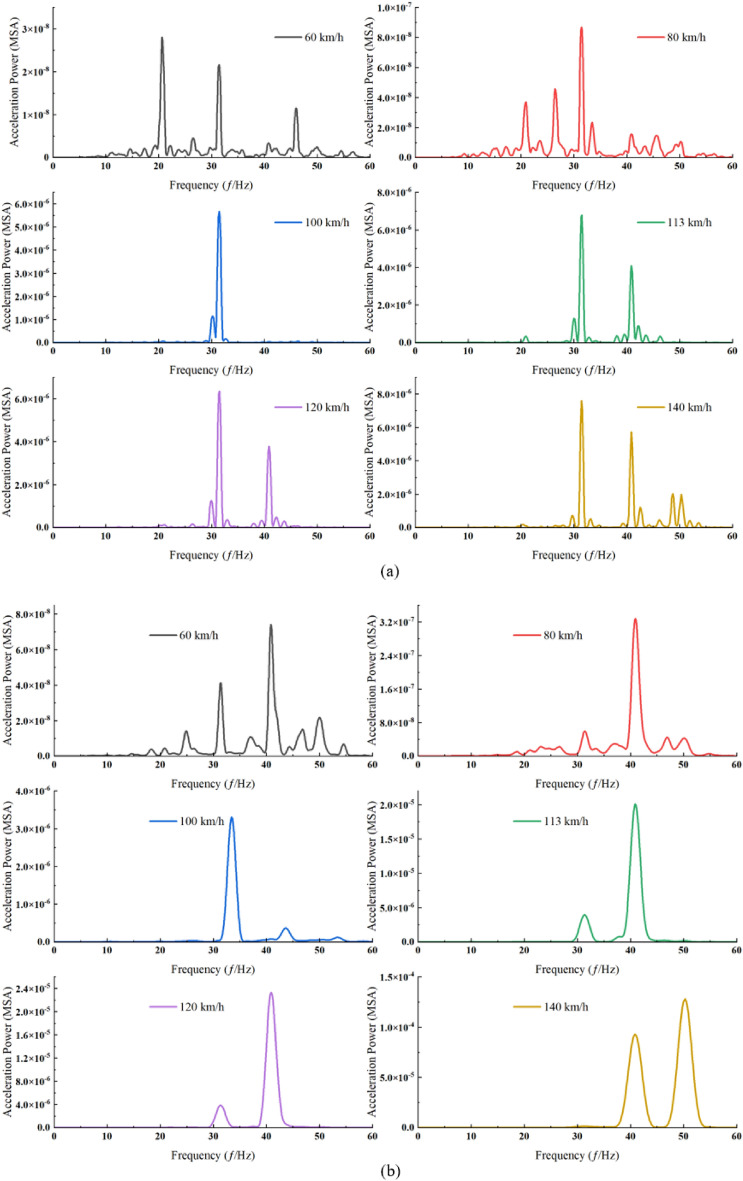

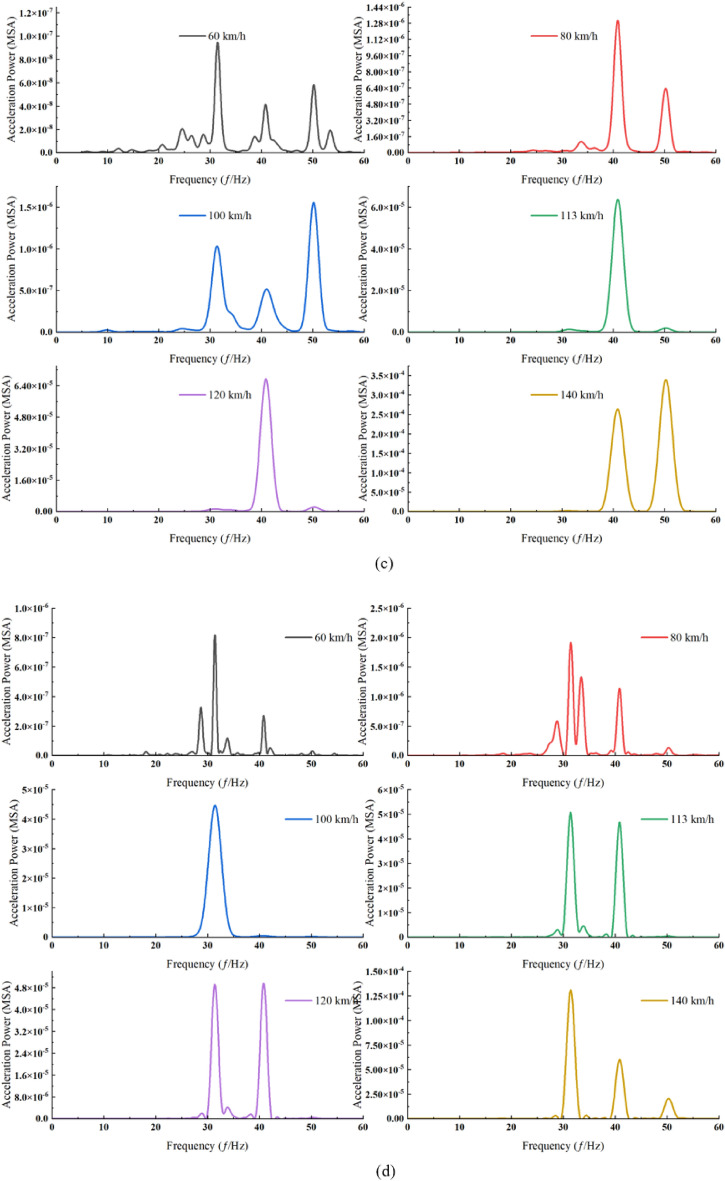

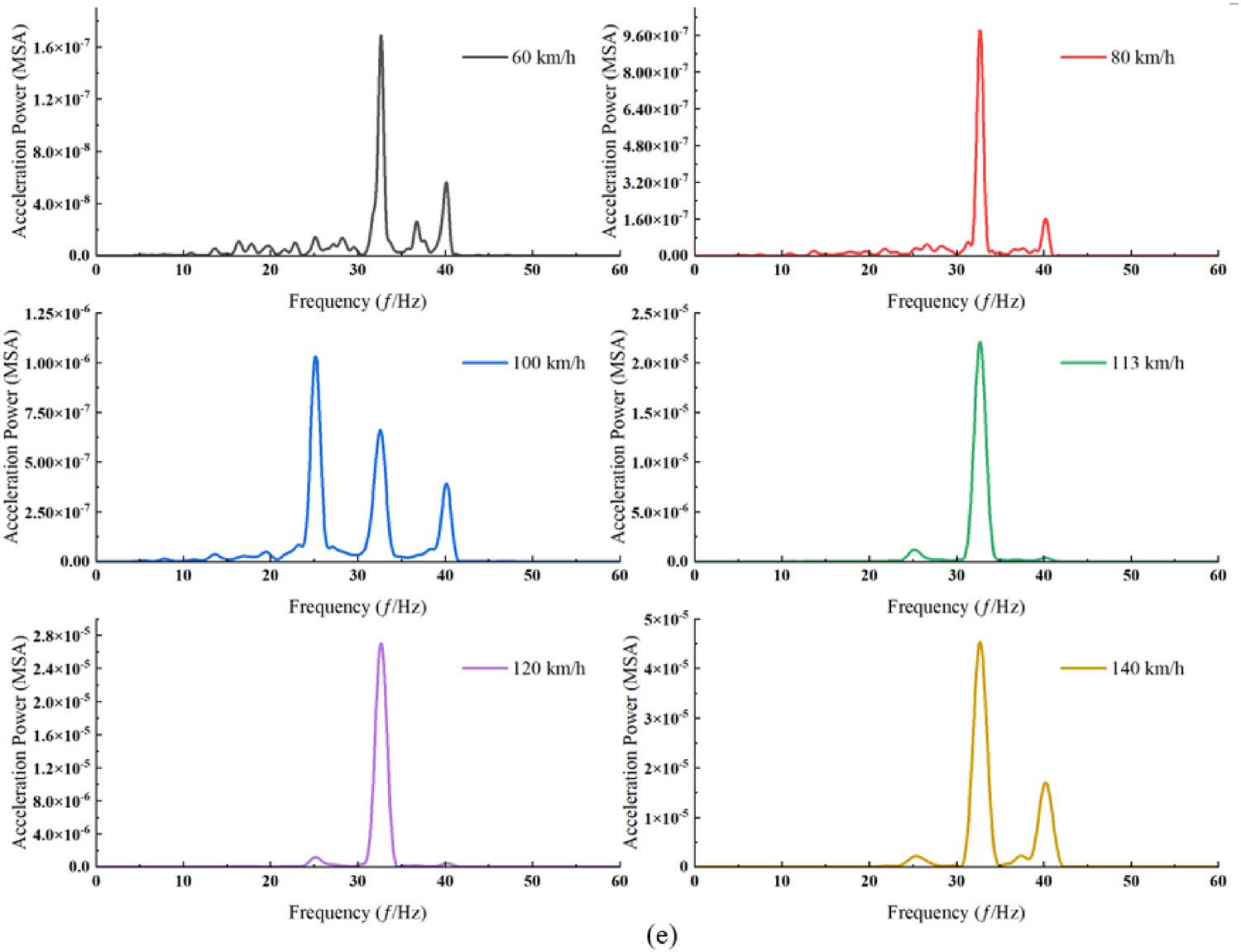
Power spectra of acceleration at each measurement point under various train speed conditions. (**a**) Measurement point P1. (**b**) Measurement point P2. (**c**) Measurement point P3. (**d**) Measurement point P4. (**e**) Measurement point P5.

At measurement point P1 (Fig. 6a), the dominant frequency rises from 21 to 32 Hz as speed increases. Within the 60–100 km/h speed range, the amplitude of the high-frequency component exhibits a gradual escalation, albeit at a slower rate compared to the intermediate-frequency component. Notably, the amplitude of the dominant frequency in the power spectrum experiences its most significant multiplication when the train speed escalates from 100 to 113 km/h. For velocities below 100 km/h, the acceleration in power amplitude within the 0–35 Hz frequency band, as speed increases, is markedly higher than in the frequency band above 35 Hz. Conversely, at velocities ranging from 100 to 140 km/h, the growth rate of power amplitude in the frequency band exceeding 35 Hz surpasses that in the band below 35 Hz. It is observed that the peak magnitude of the power spectrum remains relatively stable within the 100–140 km/h speed range. However, there is a rapid increase in the amplitude of the power spectrum for high-frequency components.

At measurement point P2 (Fig. 6b), the predominant frequency predominantly hovers near 41 Hz, with a notable decrease to 34 Hz occurring exclusively at a speed of 100 km/h. Between speeds of 80 km/h and 100 km/h, the power amplitude within the 30–35 Hz frequency band exhibits the most rapid increase. For velocities below 120 km/h, the acceleration in power amplitude with speed in the 0–45 Hz band is considerably greater than in the band above 45 Hz. Conversely, in the speed range of 120–140 km/h, the growth rate of power amplitude in the band above 45 Hz significantly surpasses that within the same frequency band.

At measurement point P3 (Fig. 6c), in the 38–44 Hz frequency band, the most rapid power increase occurs as train speed accelerates from 100 to 113 km/h. Furthermore, the greatest surge in power amplitude is noted within the 30–35 Hz band when the train speed rises from 80 to 100 km/h. Below 120 km/h, the acceleration of power amplitude with speed in the 0–45 Hz frequency band is markedly higher than in the frequency band centered at 45 Hz. Conversely, for speeds ranging from 120 to 140 km/h, the growth rate in the frequency band above 45 Hz significantly exceeds that in the band below 45 Hz.

At measurement point P4 (Fig. 6d), the dominant frequency consistently remains around 31 Hz despite increasing train speeds. With increasing speeds, a notable escalation in the high-frequency component is observed, particularly during three distinct phases: speed transitions from 60 to 80 km/h, 100 to 113 km/h, and 120 to 140 km/h. Additionally, two peaks, exhibiting closely similar amplitudes, are recorded at speeds of 113 km/h and 120 km/h, corresponding to frequencies of 31 Hz and 41 Hz, respectively. As the train speed escalates from 100 to 113 km/h, the increase in power spectral amplitude within the 39–42 Hz frequency band is substantially greater than in other frequency bands. At train speeds below 113 km/h, the acceleration of power amplitude with speed in the 0–35 Hz band is considerably more pronounced than in the frequency band centered at 35 Hz. Additionally, with train speeds rising from 113 to 140 km/h, the growth rate of power amplitude in the frequency band above 35 Hz significantly exceeds that in the band below 35 Hz.

At measurement point P5 (Fig. 6e), the dominant frequency remains constant at 33 Hz across all speeds, except 100 km/h. Notably, the most rapid increase in power amplitude occurs within the 39–42 Hz frequency band as speeds escalate from 120 to 140 km/h. Furthermore, a significant rise in the power amplitude of the 39–42 Hz band is evident when speeds are below 120 km/h. While the enhancement of the high-frequency component is not substantial at speeds under 120 km/h, it becomes pronounced with speed increments from 120 to 140 km/h.

Furthermore, it is observed that within the 60–100 km/h speed range, the amplitude of the high-frequency component exhibits a gradual increase at all measurement points, except for measurement point P3. However, this increase occurs at a slower pace when compared to the growth rate of the mid-frequency component. It is observed that the power amplitude of the low-frequency component escalates at a slower rate compared to the medium and high-frequency components as train speed rises. Concurrently, the power spectrum increasingly concentrates within specific frequency bands. This phenomenon can be attributed to the diminishing influence of environmental background vibrations on the system with rising train speeds. The observed data not only includes environmental vibrations caused by vehicle operations but also contains vibrations generated by nearby factory machinery, construction sites, and ground motion, as well as observation errors arising from observation equipment and methods. Such interfering vibrations or errors are commonly referred to as background vibrations. Notably, as train speed advances from 100 to 113 km/h and further from 120 to 140 km/h, the amplitude of the high-frequency component accelerates more markedly compared to the mid-frequency component. Hz as high frequency.

**Figure 7:**
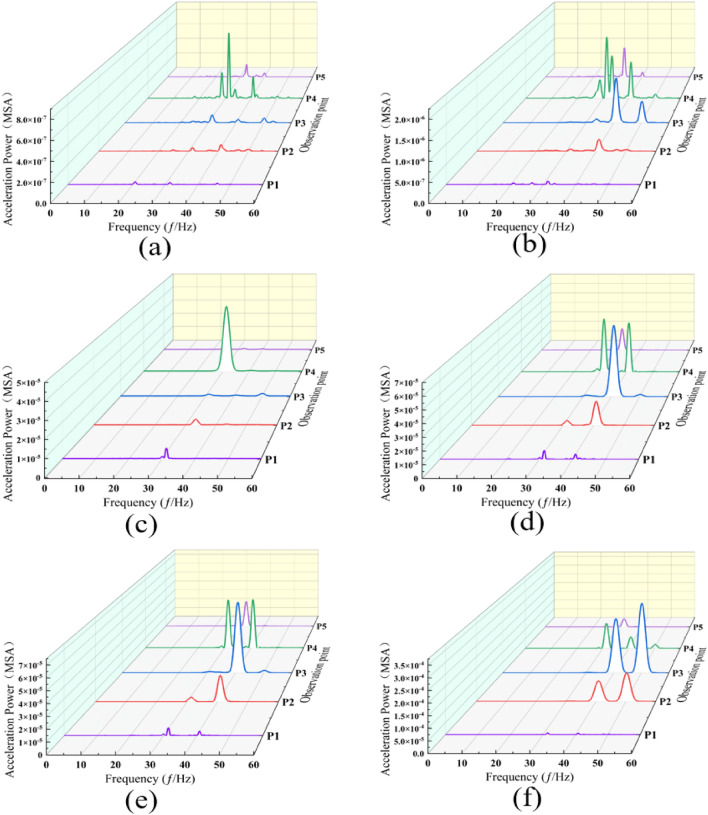
Power spectra at each measurement point across various train speeds under no load. (**a**) 60 km/h. (**b**) 80 km/h. (**c**) 100 km/h. (**d**) 113 km/h. (**e**) 120 km/h. (**f**) 140 km/h.

Through the comparison of ground surface vibration response spectra across various speeds, it is observed that below 100 km/h, the dominant frequency at measurement point P3 situated between two tunnels exhibits an upward trend, attributable to the proximity of the neighboring tunnels. Within the speed range of 80 km/h and below, there is a marked increase in the high-frequency component at specific measurement points compared to others. In the speed bracket of 100–120 km/h, power pre-dominantly focuses on the dominant frequency. A comparative analysis of the measurement points on either side of the travel tunnel reveals a notably greater escalation in both the high-frequency component and amplitude at measurement point P3 than at measurement point P5. The observed phenomenon can be attributed to the differing structural properties of the neighboring tunnels compared to the surrounding soil, which leads to the reflection of elastic waves. This effect is compounded by the reflection of near-field body waves at the ground surface, the superposition effect of their incidence, and the attenuation of body waves. Notably, as train speed increases, the prominence of this phenomenon is further accentuated (Fig. 7). Observations from measurement points P3 to P1 reveal a rapid decay in the high-frequency component. This attenuation is attributed to the significant damping effect of the soil on vertical high-frequency vibrations^51^.

#### Analysis of vibration acceleration levels at ground measurement points across varying speed conditions

**Figure 8:**
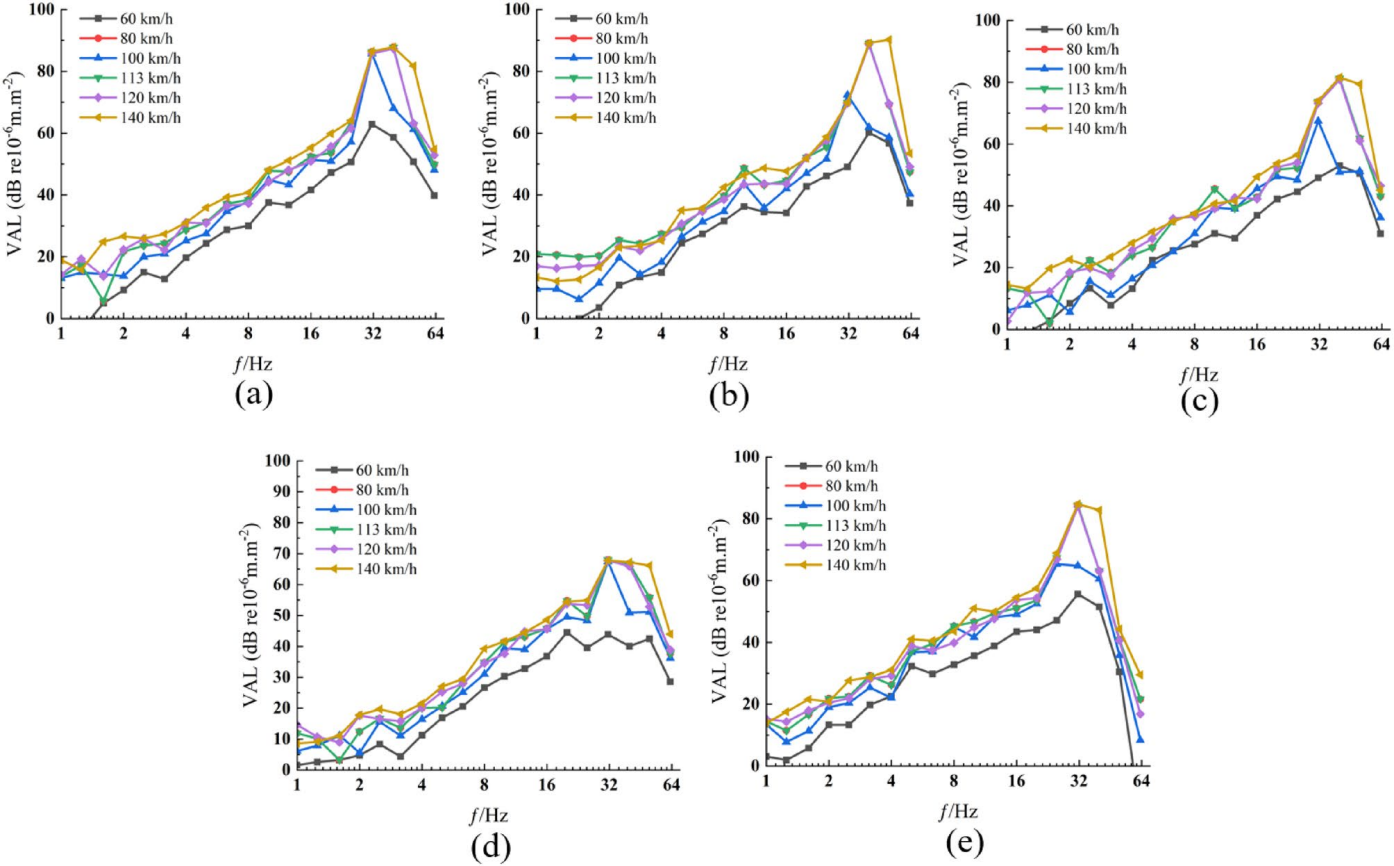
Vibration acceleration levels at each measurement point across various speeds. (**a**) Measurement point P1. (**b**) Measurement point P2. (**c**) Measurement point P3. (**d**) Measurement point P4. (**e**) Measurement point P5.

The 1/3-octave vibration acceleration levels at each measurement point were computed for various train speeds. Through comparison of vibration acceleration levels at varying speeds across each measurement point, it is observed that the acceleration level at 100 km/h is markedly lower than at 80 km/h. Additionally, at each measurement point, the vibration acceleration level begins to decrease starting from a frequency of 31.5 Hz as the train speed reaches 100 km/h (Fig. 8).

The peak vibration acceleration level at each measurement point attains its maximum at a speed of 140 km/h and registers its minimum at 60 km/h. Observations from point P4 to P1, situated above the travel tunnel, reveal that the peak vibration acceleration level escalates at 140 km/h, culminating in the highest peak at point P2.

At measurement point P5, the vibration acceleration level exhibits a rapid decay starting from 31.5 Hz. Moreover, the acceleration level at 63 Hz is considerably lower compared to other measurement points at this frequency. The primary reason for this observation is the absence of adjacent tunnels in the area stretching from measurement point P4 to P5, which would otherwise influence the vibration response.

#### Analysis of ground vibration response power spectrum due to load variation

**Figure 9:**
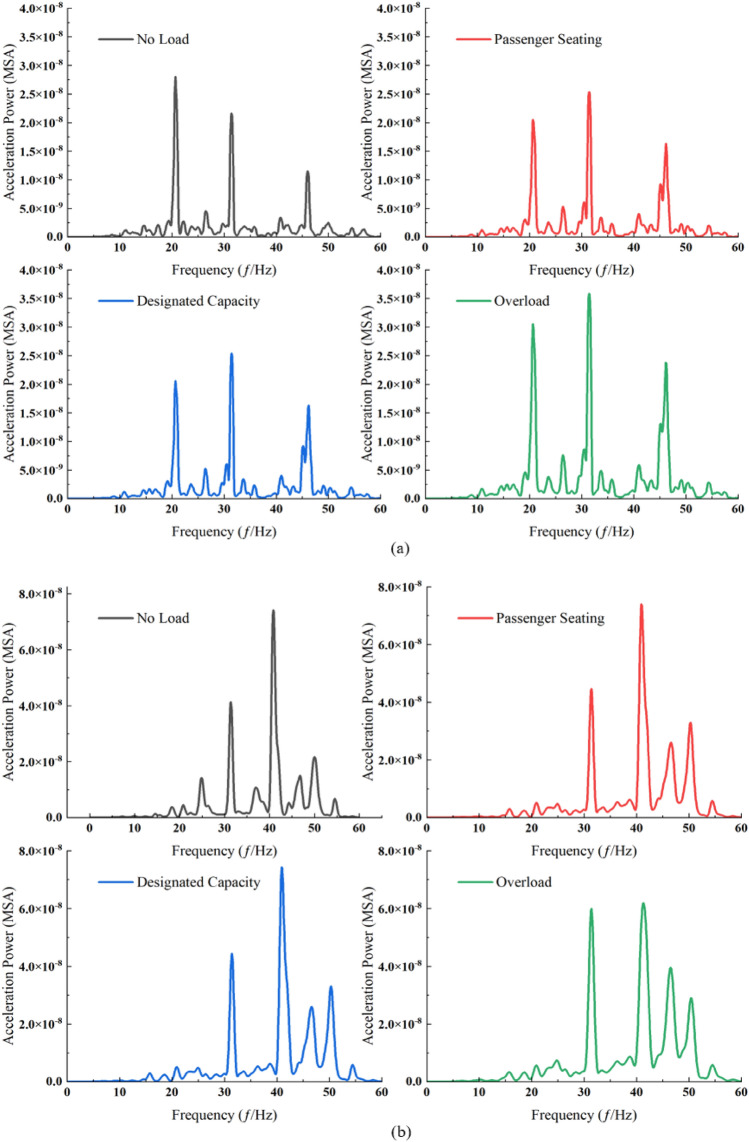

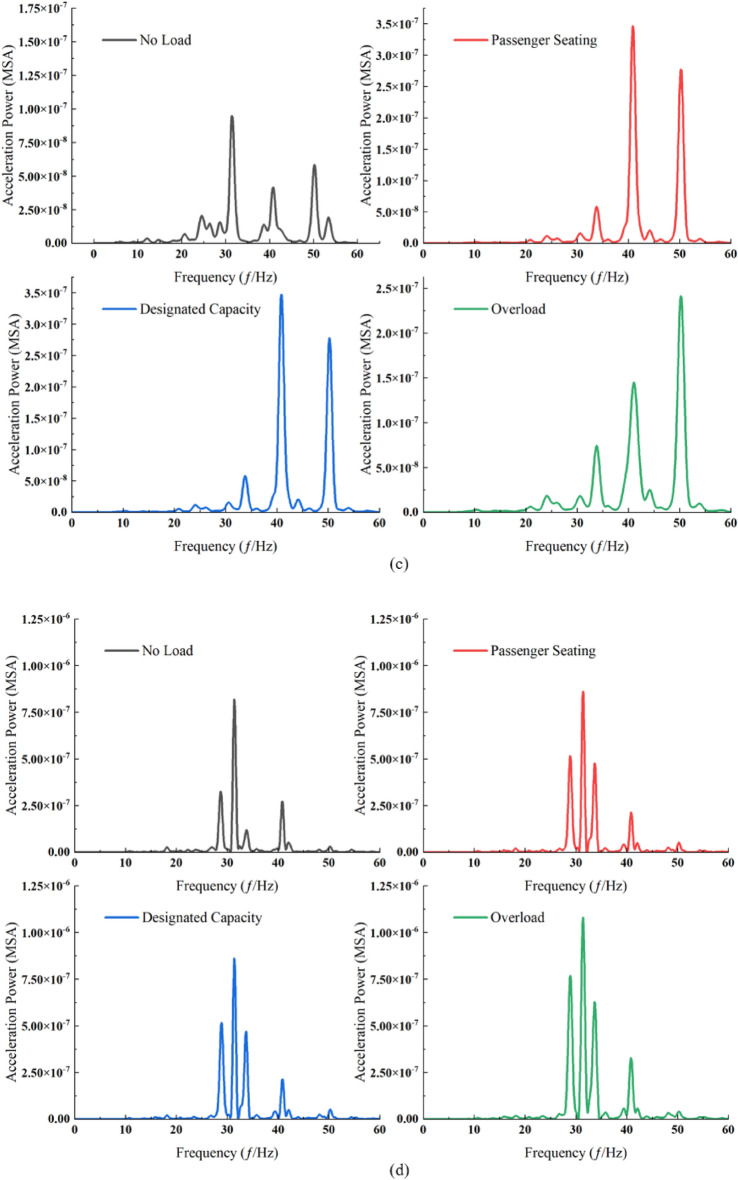

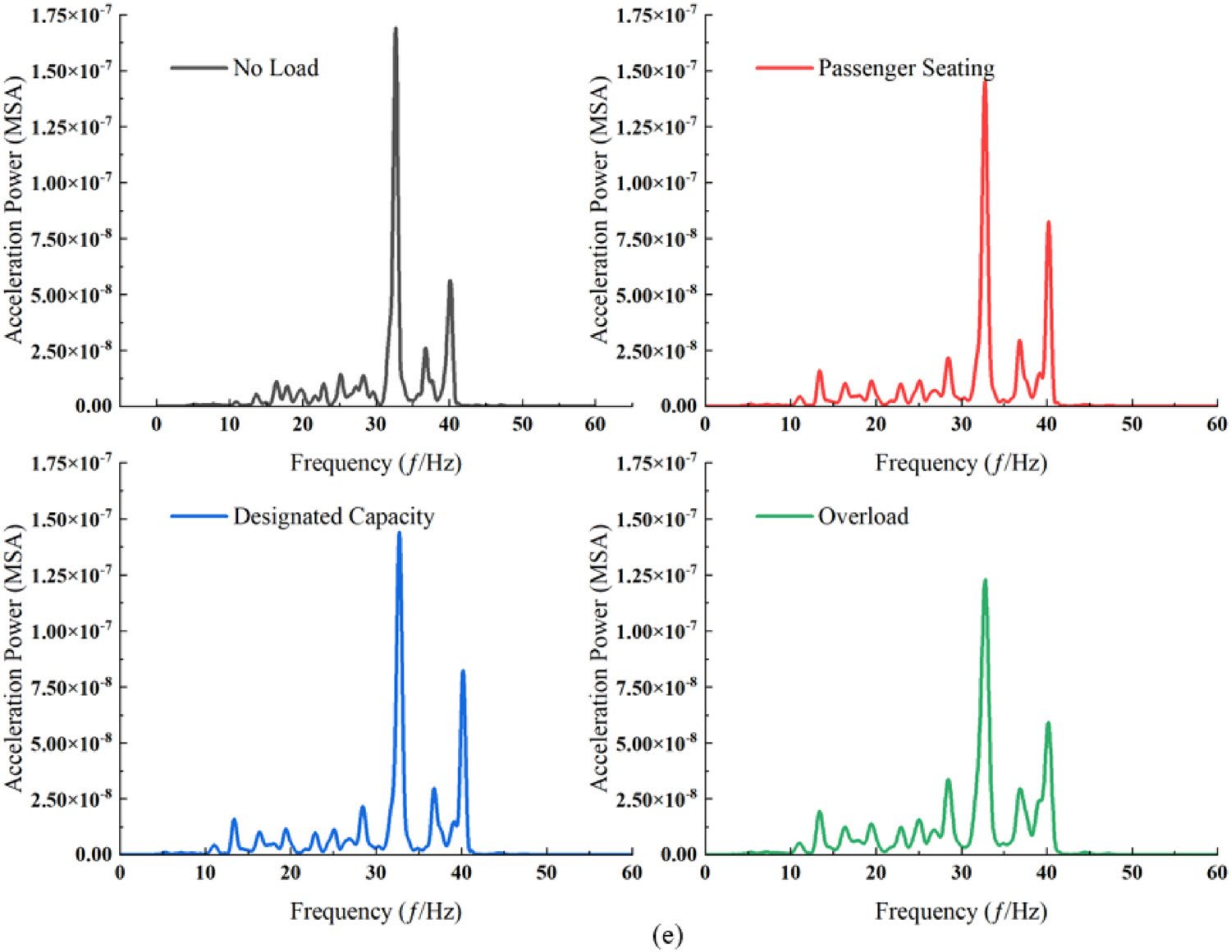
Power spectrum at ground measurement points across different load cases. (**a**) Measurement point P1. (**b**) Measurement point P2. (**c**) Measurement point P3. (**d**) Measurement point P4. (**e**) Measurement point P5.

The power spectral amplitude at each measurement point markedly escalates with the rising passenger load, particularly in the middle and high-frequency ranges (Fig. 9). This trend, however, excludes measurement points P2 and P3, where the maximum power spectral amplitude is observed under overload conditions. Concurrently, the dominant frequencies at measurement points P1, P3, and P5 exhibit an increase correlating with the heightened passenger load. Additionally, it is observed that the escalation in load has a negligible impact on the frequency band below 10 Hz.

**Figure 10:**
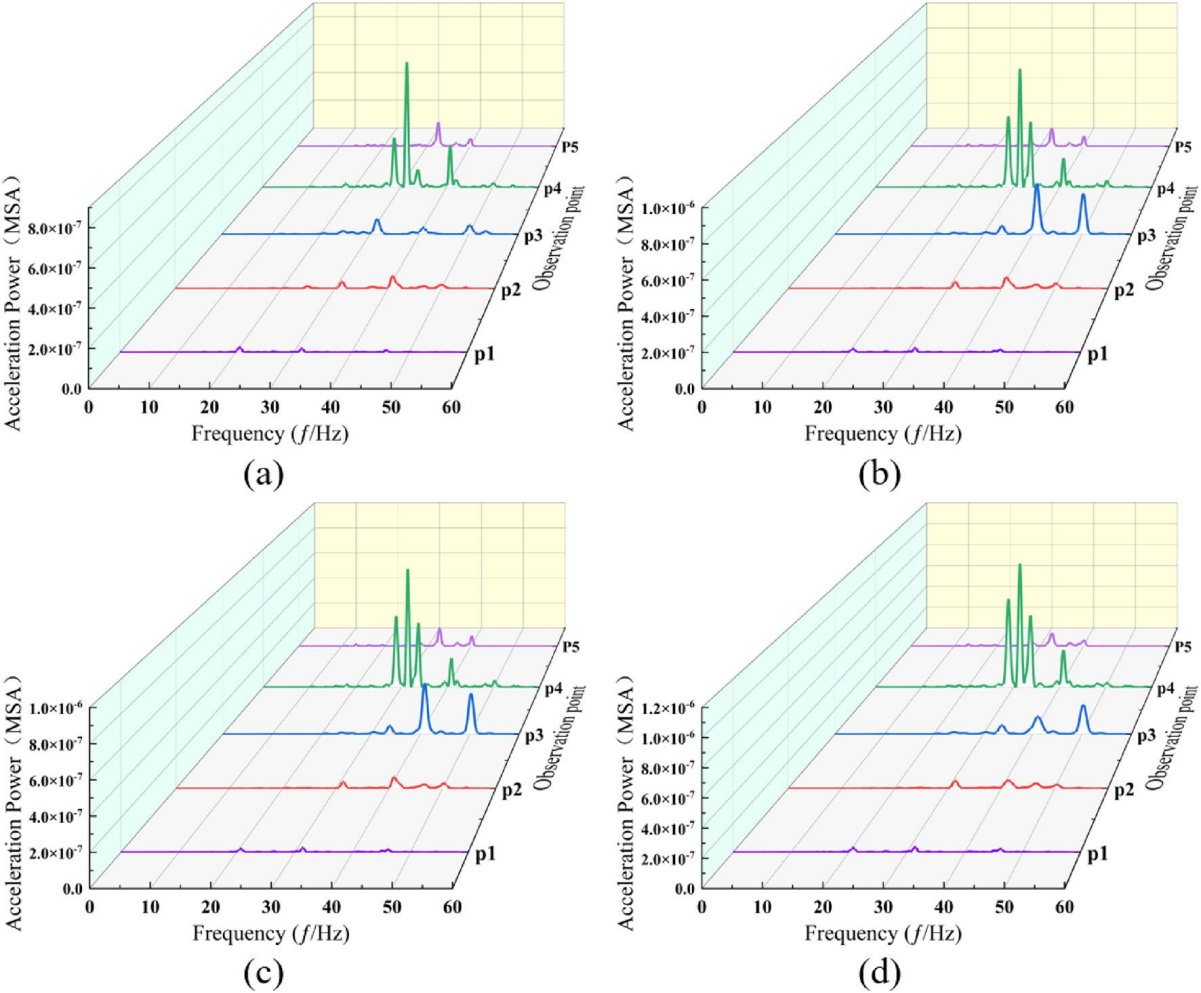
Power spectrum at ground surface measurement points for each load case. (**a**) No load conditions. (**b**) Passenger seating conditions. (**c**) Designated capacity conditions. (**d**) Overload conditions.

Under each loading condition, the ground surface vibration exhibited a distinct pattern (Fig. 10), with attenuation extending from measurement point P4 to both sides. At measurement point P3, an increase in the high-frequency component was noted in comparison to point P5, and similarly, points P2 and P1 demonstrated a higher presence of high-frequency components than point P5. This pattern mirrors the behavior observed under varying train speed conditions. When considering passenger seating and fixed occupancy, the disparities between the two power spectra are minimal, with amplitude being the primary exception. Moreover, the attenuation trend of high-frequency components from point P3 to P1 parallels the trend observed under consistent train speed conditions.

#### Time-course analysis of ground vibration response acceleration under variable speed conditions

Given the minimal impact of passenger capacity on the train, the analysis focuses solely on the acceleration time range of ground vibration response under varying train speed conditions. The maximum acceleration values at each measurement point for each speed condition are collated as shown in Table 3.

**Table 3:** Maximum acceleration values at each measurement point for various speed conditions.

Observation point (km/h)	P1 (m/s^2^)	P2 (m/s^2^)	P3 (m/s^2^)	P4 (m/s^2^)	P5 (m/s^2^)
60	0.0076	0.0156	0.0087	0.0114	0.0072
80	0.0065	0.0088	0.0094	0.0202	0.0073
100	0.0220	0.0203	0.0195	0.0765	0.0143
113	0.0287	0.0346	0.0562	0.1067	0.0357
120	0.0295	0.0315	0.0514	0.1013	0.0329
140	0.0295	0.0354	0.0485	0.0942	0.0354

**Figure 11:**
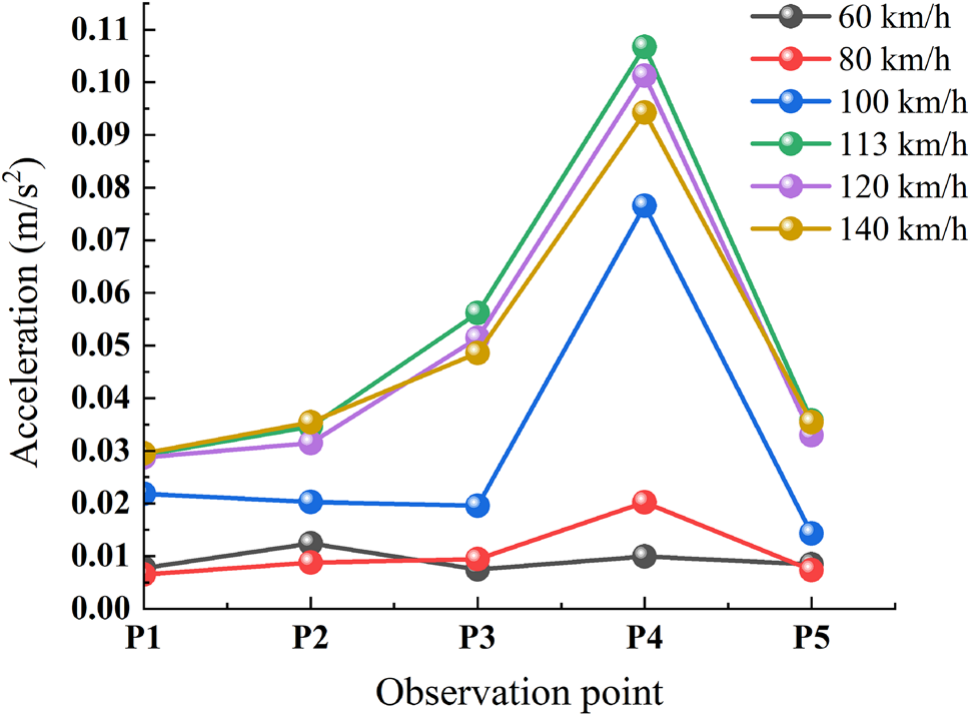
Maximum vibration acceleration at ground surface measurement points for various speed conditions.

Figure 11 illustrates that the maximum acceleration at each measurement point is less pronounced at train speeds of 80 km/h or lower. Conversely, as train speed surpasses 80 km/h, there is a rapid increase in acceleration amplitude, peaking at a speed of 113 km/h. Beyond 120 km/h, the maximum acceleration value shows a declining trend. Simultaneously, it is noted that the attenuation amplitude between measurement points P2 and P1 is considerably less than that observed from P3 to P2. Moreover, the attenuation amplitude from measurement point P4 to adjacent point P3 is markedly less than that from P4 to adjacent point P5.

## Discussion

Through research, we can find that the increase in vehicle speed causes an increase in the high-frequency components of the power spectrum and the amplitude of the power spectrum. This phenomenon is particularly obvious when the vehicle speed exceeds the Rayleigh wave speed. The increase in train load has a significant impact on the amplitude of the power spectrum but does not have much impact on the frequency component. This is because the load has a small impact on the frequency component of the train load. In addition, there is a certain amplification trend in the peak acceleration and power spectrum of the measuring point in the middle of adjacent tunnels. This is because the properties of adjacent tunnels are different from those of the surrounding soil, resulting in certain reflections of elastic waves. The reflection of near-field body waves on the ground surface, the incident superposition effect, and the attenuation effect of body waves are superimposed on each other to form this phenomenon. Especially as the vehicle speed increases, this phenomenon becomes more obvious.

## Conclusion

This paper utilizes geological survey data from urban railways to construct a tunnel–soil 3D finite element dynamic coupling model. The reliability of the model is validated using field observation data. Subsequently, we analyzed the influence of train speed and passenger capacity on the ground surface vibration response and propagation characteristics of the urban railway underground section. The ground vibration characteristics were analyzed at different vehicle speeds and loads, leading to the following conclusions:

1. As the vehicle speed increases, the prominent frequencies observed at the two measuring points adjacent to the adjacent tunnel surpass those observed at the measuring points above the driving tunnel. The power amplitude near the prominent frequency of each measuring point gradually increases with the vehicle speed, yet the power spectrum amplitude of the measuring point above the driving tunnel exhibits less influence when the vehicle speed ranges from 100 to 120 km/h. The power spectrum amplitude of the frequency band ranging from 39 to 42 Hz at the measurement point above the driving tunnel exhibits the most rapid increase as the vehicle speed escalates from 100 to 113 km/h. Additionally, compared to other vehicle speeds, the frequency band where the vibration acceleration level experiences significant attenuation at each measuring point expands notably when the vehicle speed reaches 100 km/h.
2. When the vehicle speed increases from 100 to 113 km/h and from 120 to 140 km/h, the growth amplitude of the high-frequency component significantly outpaces that of the mid-frequency component. In the vehicle speed range from 60 to 100 km/h, the amplitude of the high-frequency component at the measurement points, except for the one between the two tunnels, gradually increases. However, compared to the mid-frequency component, the rate of increase is slightly slower. As the speed increases, the power spectrum gradually concentrates within specific frequency bands. This trend occurs because, with the escalating vehicle speed, the influence of environmental background vibration diminishes progressively.
3. Due to the differing structural properties between adjacent tunnels and the surrounding soil, elastic waves will be partially reflected. The effects of reflection and incidence superposition of near-field body waves on the ground surface, along with the attenuation of body waves, combine. The high-frequency component of the power spectrum at measuring points between adjacent tunnels is notably higher than that of other measuring points. This phenomenon becomes more pronounced with increasing vehicle speed.
4. The power spectrum amplitude of each measuring point increases with the rise in passenger capacity, particularly with a significant increase observed in the amplitude of the mid-and high-frequency components. The power spectrum amplitude of the measuring point above the traffic tunnel reaches its maximum when overloaded. With an increase in passenger volume, the amplitude of the high-frequency component of the power spectrum at measuring points between the two tunnels experiences a significant increase.
5. When the vehicle speed is 80 km/h and below, it has little effect on the peak acceleration of each measuring point. However, once the vehicle speed surpasses 80 km/h, the acceleration amplitude of the measuring point above the driving tunnel rises rapidly, reaching a peak at a vehicle speed of 113 km/h before gradually decreasing.
6. When the train passes through an area with strict control over the acceleration vibration level at a certain frequency and acceleration is needed, the impact generated at a speed of 100 km/h is significantly smaller than at other speeds. On the other hand, when the train passes through an area with strict control over the maximum acceleration and acceleration needed, the impact generated at a speed of 80 km/h is not significantly different from that at a speed of 60 km/h. Additionally, when further increasing the speed, it is advisable to avoid reaching a speed of 113 km/h if possible.
7. When the train speeds are 80 km/h, 113 km/h, and 120 km/h, the acceleration vibration levels are relatively similar. However, when the speed reaches 140 km/h, there is an overall upward trend in the acceleration vibration levels. When the train speeds are 80 km/h, 113 km/h, and 120 km/h, the acceleration vibration levels are relatively close. However, when the train speed reaches 140 km/h, the acceleration vibration levels overall show an upward trend. Therefore, when passing through areas with strict requirements on certain frequency bands, the train speed should be kept below 120 km/h.

## Data Availability

The data that support the findings of this study are available from the corresponding author, [F.T. Wang], upon reasonable request.

## References

[1] Zhang H, Yuan J (2020). Analysis of the effect of subway operation on the vibration of buildings along the line based on edge computing. IEEE Access.

[2] Yin JB, Cao XY, Huang XY (2021). Association between subway and life satisfaction: Evidence from Xi’an, China. Transp. Res. D Transp. Environ..

[3] Shi YB (2023). Research on anti-terrorism emergency response in China’s subway transportation. J. Ppl. Pub. Secy. Uni. Chn..

[4] Xia, H. *Traffic Induced Environmental Vibrations and Controls.* (New York, 2010).

[5] Ma J, Li CJ, Kan M, Chai YW (2018). A multilevel analysis of perceived noise pollution, geographic contexts, and mental health in Beijing. Int. J. Environ. Sci. Technol..

[6] Zou C, Zhu RJ, Tao ZY, Quyang DQ, Chen YK (2020). Evaluation of building construction-induced noise and vibration impact on residents. Sustainability.

[7] Connolly DP, Marecki GP, Kouroussis G, Thalassinakis I, Woodward PK (2016). The growth of railway ground vibration problems—A review. Sci. Total Environ..

[8] Chen JG, Xia H, Chen SL (2010). Investigation on running-train-induced ground vibrations near railway. Eng. Mech..

[9] Zhang Z, Ma F, Zhang B (2018). Vibration measurement of long-span floors in high-speed railway station. J. Vibroeng..

[10] Tekergul, E., Zulfikar, A. C., Celebi, E., Kirtel, O. & Goktepe, F. Effect of trench barrier on free field motion due to the train and highspeed train passages. In *7th ECCOMAS Thematic Conference on Computational Methods in Structural Dynamics and Earthquake Engineering (COMPDYN 2019)* 862–870 (2019). 10.7712/120119.6964.19813.

[11] Xu R, Li X, Yang W, Rabiei M, Yan C, Xue S (2019). Field measurement and research on environmental vibration due to subway systems: A case study in eastern China. Sustainability.

[12] He YL, Xiang Y (2012). Test and analysis of environmental vibration of Weinan north elevated station of Zhengzhou-Xi’an High-Speed Railway. Noise Vib. Control.

[13] Wang FT, Tao XX, Cui GH (2011). Test in situ for free ground vibration near urban railway line. J. Vib. Shock.

[14] Boogaard MA, Li Z, Dollevoet RPBJ (2018). In situ measurements of the crossing vibrations of a railway turnout. Measurement.

[15] Gupta S, Degrande G, Lombaert G (2009). Experimental validation of a numerical model for subway induced vibrations. J. Sound Vib..

[16] Lou ML, Li SJ (2007). Evaluation of buildings’ vibration induced by underground trains. J. Vib. Shock.

[17] With C, Bahrekazemi M, Bodare A (2006). Validation of an empirical model for prediction of train-induced ground vibrations. Soil Dyn. Earthq. Eng..

[18] Guo T, Cao Z, Zhang Z (2017). Numerical simulation of floor vibrations of a metro depot under moving subway trains. J. Vib. Control.

[19] Lin G, Liu D (2023). Numerical simulation of train-induced structural vibration. J. Phys. Conf. Ser..

[20] Wang S, Cao Z, Yifei Xu (2022). Prediction and mitigation of train-induced vibrations of over-track buildings on a metro depot: Field measurement and numerical simulation. J. Vib. Control.

[21] Fiala P, Degrande G, Augusztinovicz F (2007). Numerical modelling of ground-borne noise and vibration in buildings due to surface rail traffic. J. Sound Vib..

[22] Kouroussis G, Conti C, Verlinden O (2013). Investigating the influence of soil properties on railway traffic vibration using a numerical model. Veh. Syst. Dyn..

[23] Huang Q, Li P, Zhang D (2021). Field measurement and numerical simulation of train-induced vibration from a metro tunnel in soft deposits. Adv. Civ. Eng..

[24] Ropars, P., Vuylsteke, X. & Augis, E. Vibrations induced by metro in sensitive buildings; Experimental and numerical comparisons. In *11th European congress and exposition on noise control engineering; 2018 May 27–31; Crete, Greece* 381–1386 (European Acoustics Association, 2018). http://refhub.elsevier.com/S0267-7261(22)00060-4/sref9.

[25] Lopes P, Costa PA, Ferraz M (2014). Numerical modeling of vibrations induced by railway traffic in tunnels: From the source to the nearby buildings. Soil Dyn. Earthq. Eng..

[26] Zhu ZH, Wang LD, Costa PA (2019). An efficient approach for prediction of subway train-induced ground vibrations considering random track unevenness. J. Sound Vib..

[27] Smith IM, Griffiths DV (1988). Programming the Finite Element Method.

[28] Petyt M (2010). Introduction to Finite Element Vibration Analysis.

[29] Liu GG, Quek SS (2003). The Finite Element Method: A Practical Course.

[30] Ding Z, Li DW (2019). Dynamic response analysis on vibration of ground and track system induced by metro operation. Eng. Comput..

[31] Xiong YL, Zhang SL, Wu ZZ (2020). Comparative analysis of the influence of building vibration on human comfort caused by blasting and subway train loads. IOP Conf. Ser. Earth Environ. Sci..

[32] Zhou YF, Bai XX, Wang SN (2022). Structural vibration analysis of historical drum tower structure caused by underground train running. Front. Earth Sci..

[33] Ma XL, Ba ZN, Gao YH (2019). Vibration effect of metro operation on buildings along Tianjin Binhai New Area in soft soil areas. Chin. J. Geotech. Eng..

[34] Tian T, Lei Y, Qi FL (2018). Vibration response transmission of lining arch due to train speed-changing vibration load. Eng. Mech..

[35] Liu YW, Ba ZN, Gao YH (2019). Influences of metro operation on ground vibration along Tianjin Z2 line. Chin. J. Geotech. Eng..

[36] Zhang YE, Bai BH, Zhang YQ (2006). Influence of burial depth on the vibration response of trains in subway interval tunnels. J. Vib. Shock.

[37] Xu R, Li XC, Cheng H (2016). Numerical prediction of ground vibration induced by subway based on vibration source and propagation model. J. Arch. Civ. Eng..

[38] Verhas HP (1979). Prediction of the propagation of train-induced ground vibration. J. Sound Vib..

[39] Kurzweil LG (1979). Ground-borne noise and vibration from underground system. J. Sound Vib..

[40] Wang Li, Wang P, Wei K (2022). Ground vibration induced by high speed trains on an embankment with pile-board foundation: Modelling and validation with in situ tests. Transp. Geotech..

[41] Gaoa G, Yao S, Yang J (2019). Investigating ground vibration induced by moving train loads on unsaturated ground using 2.5D FEM. Soil Dyn. Earthq. Eng..

[42] Li JS, Li KC (1995). Finite element analysis of dynamic response of High-Speed Railway subgrade. J. Chin. Railw. Soc..

[43] Liang B, Cai Y (1999). Dynamic analysis on subgrade of High-Speed Railways in geometric irregular condition. J. Chin. Railw. Soc..

[44] Jenkins H (1974). The effect of track and vehicle parameters on wheel/rail vertical dynamic loads. Railw. Eng..

[45] Pan CS, Pande GN (1984). Finite element dynamic load response of train in loess tunnel preliminary numerical determination and analytical study. Chin. Civ. Eng. J..

[46] Liao ZP (2002). Introduction to Wave Motion Theories in Engineering.

[47] Du XL (2009). Theories and Methods of Wave Motion for Engineering.

[48] Ma HW, Wu B (2000). Elastic Dynamics and Its Numerical Methods.

[49] Zhang QL, Liu LY, Li JY (2015). Numerical analysis of ground vibration amplification phenomenon caused by subway operation. Noise Vib. Control.

[50] GB 50490. *Technical specifications for urban rail transit.* (China, 2009).

[51] Zong G, Zhang YH, Ren XS (2017). In-situ measurement and mechanism analysis for local amplification phenomena of metro-induced ground-borne vibration. J. Vib. Shock.

